# Defining and deconstructing girl child marriage and applications to global public health

**DOI:** 10.1186/s12889-020-09545-0

**Published:** 2020-10-15

**Authors:** Yvette Efevbera, Jacqueline Bhabha

**Affiliations:** 1 Department of Global Health and Social Medicine, Harvard Medical School, 641 Huntington Ave, Boston, MA 02115 USA; 2 Department of Global Health and Population, Harvard T. H. Chan School of Public Health, 665 Huntington Ave, Bldg. 1, 11th floor, Boston, MA 02115 USA; 3 FXB Center for Health and Human Rights, Harvard T. H. Chan School of Public Health, 651 Huntington Ave, 7th Floor, Boston, MA 02115 USA

**Keywords:** Child marriage, Child, Definition, Girl, Health, Marriage

## Abstract

An estimated 650 million girls and women alive today married before their 18th birthday. Referred to as girl child marriage, the formal or informal union of the girl-child before age 18, the practice is increasingly recognized as a key roadblock to global health, development, and gender equality. Although more research than ever has focused on girl child marriage, an important gap remains in deconstructing the construct. Through an extensive review of primary and secondary sources, including legal documents, peer-reviewed articles, books, and grey literature across disciplines, we explore what the term “girl child marriage” means and why it more accurately captures current global efforts than other terms like early, teenage, or adolescent marriage. To do this, we dive into different framings on marriage, children, and gender. We find that there has been historical change in the understanding of girl child marriage in published literature since the late 1800s, and that it is a political, sociocultural, and value-laden term that serves a purpose in different contexts at different moments in time. The lack of harmonized terminology, particularly in the global public health, prevents alignment amongst different stakeholders in understanding what the problem is in order to determine how to measure it and create solutions on how to address it. Our intent is to encourage more intentional use of language in global public health research.

## Background

Worldwide, an estimated 650 million girls and women alive today married before their 18th birthdays [1]. One in three girls in developing countries is married before age 18, while one in five girls is married before age 15 [2]. Referred to as girl child marriage, the formal or informal union of the girl-child before age 18, the practice is increasingly recognized as a key roadblock to global health, development, and gender equality. South Asia and sub-Saharan Africa regions account for the largest number of women married as children [1]; however, a recent report also shows high rates in previously understudied geographies like South America, where 25% of girls married before age 18 [3]. Girl child marriage is also increasingly documented in high-income countries like the United States, where a recent study estimates nearly 1% of 15–17-year-olds surveyed had been married, with variation across states [4]. (See Table 1 for countries with the highest prevalence rates.) Despite increasing global consensus that girl child marriage should be prevented given its harms to the rights and well-being of girls [5–7], no region is on track to achieve Sustainable Development Goal (SDG) 5 Target 3 to eliminate all harmful practices including child, early, and forced marriage [1].

**Table 1:** 20 countries with the highest prevalence of girl child marriage among 20 to 24-year-old women^a^

Country	Married by 15 (%)	Married by 18 (%)
Niger	28	76
Central African Republic	29	68
Chad	30	67
Bangladesh	22	59
Burkina Faso	10	52
Mali	17	52
South Sudan	9	52
Guinea	19	51
Mozambique	14	48
Somalia	8	45
Nigeria	18	44
Malawi	9	42
Madagascar	12	41
Eritrea	13	41
Ethiopia	14	40
Uganda	10	40
Nepal	7	40
Sierra Leone	13	39
Democratic Republic of the Congo	10	37
Mauritania	18	37

^a^ Percentages are reported in the March 2018 update of the UNICEF Global Databases for Child Marriage

Although more research than ever has focused on girl child marriage, an important gap remains in deconstructing the construct. Girl child marriage elaborates on the definition for child marriage, often synonymously referred to as early marriage, which is defined by the United Nations Children’s Fund (UNICEF) as the union of an individual before age 18 [1, 8]. Such terms have become normative, used today among governments, non-governmental organizations, advocacy groups, popular media, researchers, and members of affected communities alike. Indeed, references to child marriage appear more frequently than a decade ago. A new Google alert regularly shares at least one new article or report on child marriage, overwhelmingly focused on the girl-child, across disciplines including global health, public health, education, and social sciences broadly. Even popular media outlets in the United States (U.S.) such as National Public Radio (NPR), the *New York Times*, and *Teen Vogue* have increasingly discussed this social phenomenon, describing its occurrence and consequences for women all over the world [9–12].

Through an extensive review of primary and secondary sources, including legal documents, peer-reviewed articles, books, and grey literature across disciplines, we explore how the term “child marriage” has been used and defined and why “girl child marriage” more accurately captures current global efforts than other terms like early, teenage, or adolescent marriage. To do this, we dive into different framings on marriage, children, and gender. Unlike much global public health research that defines child marriage, or girl child marriage, in a single sentence, we demonstrate that it requires deconstruction. Our intent is to encourage more intentional use of language in global public health research.

## Main text

### A historical journey on terminology

The concept of the term “child marriage” appears to have strong roots in India, perhaps unsurprisingly, as it is a country with high rates that has led rigorous activism for over a century. Some of the earliest discussions identified in published literature came from India, raising questions about marital unions that were early and with questions surrounding an ability to consent [13, 14]. In a letter to an American friend in the nineteenth century, Roy [13] described critical arguments for why child marriage in Hindu culture existed at the time, suggesting thousands of years of history to reconcile. He interchangeably referred to “child marriage” and “infant marriage,” and though he never defined either term, he reported that 10% of girls and 3% of boys age 8 and below were married at his time of writing. Yet at the same time, Roy explained that the age of consent for marriage in England was similarly low – 12 years for girls and 14 years for boys – a considerable departure from England’s majority age of 21 at the time [13]. This early published reference serves as an important reminder that marriages at young ages were practiced worldwide, in Western and non-Western countries, in economically advantaged and disadvantaged countries, alike. It would not be until the Child Marriage Restraint Act of 1929 that a legal framework for reconsidering age at marriage laws was formalized in India, eventually leading to a revised Act that outlawed marriage of girls under 18 years and boys under 21 years in 1978 [15].^1^

The earliest references to the term “child marriage” in scientific articles in PubMed, a leading database for health-related research, emerged in 1955 and 1957, in the context of Israel and England respectively [17, 18]. There were no publications again until 1978, and the very limited articles focused on India, neighboring South Asian countries, and the merits of preventing child marriage for population control. The first PubMed mention of child marriage in sub-Saharan Africa was an article in 1984, which hypothesized (though did not test) adolescent sexual exposure, heightened by the common practice of child marriage, may contribute to cervical cancer [19]. Published health-related research on child marriage remained sparse over the few decades, with a slight increase in published research in 1995 (particularly in the context of India).

This slight increase in research in the mid-1990s was consistent with increasing discussions about protecting girls’ and women’s rights and promoting their sexual and reproductive health [20]. The World Summit for Children had convened world leaders for the first time around basic protections for boys and girl in 1990. The International Conference on Population Development (ICPD) in Cairo called to advance gender equality and women’s empowerment as part of a broader agenda on population growth and development in 1994 [20]. And, just one year later, the Fourth World Conference on Women in Beijing built on the momentum of the ICPD and the Beijing Declaration and the Platform for Action, an agenda for gender equality, was unanimously adopted by 189 countries. These global moments, which referenced the girl-child and marriage in their recommendations, were complemented by domestic activism in India, where much child marriage research was coming from in 1995; India had signed onto key international instruments establishing children’s and women’s rights and demographic surveys showing high rates of marriage prompted calls for marriage registration reform around this time [21].

By 2000, leading international organizations based in Europe and the U.S. promoted attention toward setting and enforcing a minimum marital age of 18 years, favoring the term “early marriage” in reference to this practice among both girls and boys [15, 22, 23]. They called for a human rights-based approach toward protecting young people, particularly girls, from early unions, which were increasingly recognized as harmful. For example, early marriage was identified as a key advocacy issue by the Forum on Marriage and the Rights of the Children, which built a global network of organizations that collectively called for increased attention to this “relatively neglected area” [23]. Similarly, the UNICEF Innocenti Center for Research [15] called for increased attention by UNICEF and other international organizations to early marriage, its harmful impacts, and solutions; they favored the term “early marriage,” sometimes interchanging it with “child marriage,” and furthered support for defining it as a union by age 18 under statutory and customary law. The International Center for Research on Women (ICRW) [22] and researchers such as Jensen and Thornton [24] also referred to early marriage as occurring before age 18 in line with international convention; their analyses looked not only at an age 18 cut-off but more broadly at trends in age at marriage and its health and well-being correlates, such as sexual and reproductive health, pregnancy, HIV/AIDS, and education.

In the few years that followed, “child marriage” gained traction as the dominant term. Technical consultations across organizations took place, UNICEF and other partners agreed to measure five indicators to better understand this construct in 2003, and by 2005, UNICEF introduced an expanded definition of child marriage [25]. In their report titled “Early Marriage: A Harmful Traditional Practice,” UNICEF used the term “child marriage” more frequently throughout the text, stating: “The term ‘child marriage’ will be used to refer to both formal marriages and informal unions in which a girl lives with a partner as if married before the age of 18” [25]. While there was not yet consensus on the inclusion of informal unions in the definition, others including the International Planned Parenthood Federation (IPPF), the Forum, and UNFPA similarly articulated a focus on “child marriage” (synonymous to “early marriage”), maintaining the age 18 threshold due to a human rights agenda and its health, social, and economic costs [26]. Publications on “child marriage” increased over time, and Raj appears to be the first to publish using the term “girl child marriage” in her paper on child marriage and health in 2010 [27]. Though she provided no rationale for her use of this revised terminology, it makes sense as a framing tool, given her research focus on the consequences for only women married as children.^2^

**Fig. 1:**
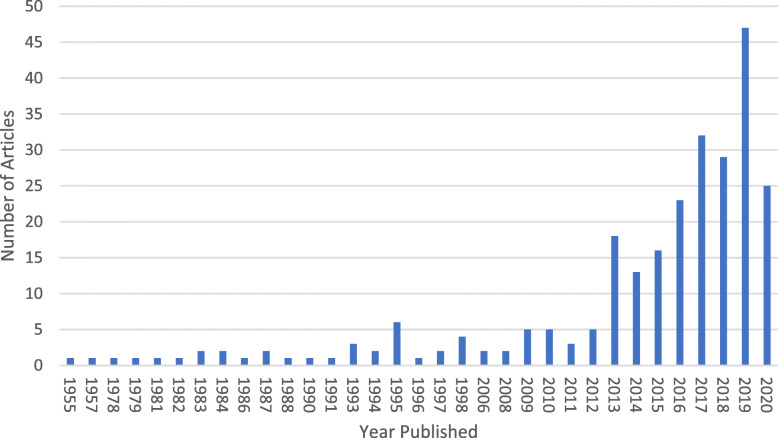
Number of PubMed articles searchable using “child marriage” published, by year

Since that time, publications on child marriage, particularly for the girl-child and as related to health, have grown exponentially. In 2019 alone, there were 47 publications in PubMed, a 25% increase from the previous year (See Fig. 1). This increase in scholarly research is accompanied by global momentum over the last decade, including: the establishment of the high profile *Girls Not Brides: The Global Partnership to End Child Marriage*, the inaugural UN International Day of the Girl featuring a call against child marriage, and the inclusion of child marriage indicators to monitor in the 2030 Sustainable Development Goals blueprint.

### Parsing out “marriage”

To understand what is referred to as “girl child marriage” requires an understanding of the construct of marriage itself. International legal frameworks have sought to define marriage. As illustrated by the Convention to Regulate Conflicts of Laws in the Matter of Marriage, drafted by 12 European countries in 1902 (and later dissolved), and the Havana Convention on Private International Law, drafted by 15 Latin American countries in 1928 [28], more than 100 years of international legal agreements have sought to legally define marriage and the rights it guarantees individuals in marriage. The 1926 Supplementary Convention on the Abolition of Slavery, the Slave Trade, and Institutions and Practices similar to Slavery also sought to ensure all individuals’ freedoms including that of women and children [29], creating foundations where marriage could only occur with each parties’ consent. These can be viewed as predecessors to understanding modern legal constructs around the global definition of marriage, and more specifically child marriage and forced marriage.

Today, legally-binding international conventions and treaties illustrate a global consensus on the rights and protections humans should be offered, including as related to a legal construct of marriage. The 1948 Universal Declaration on Human Rights provided the first internationally-agreed upon modern legal definition of marriage. Article 16 specifies that all “men and women of full age … have the right to marry and found a family,” that marriage is a union that can be formed with “free and full consent” of participants as well as dissolved, and, by interpretation, that there are rights and protections afforded to married individuals [30]. The 1962 Convention on Consent to Marriage, Minimum Age for Marriage, and Registration of Marriages reiterates the 1948 guidelines and explicitly calls for registration of marriages and eliminating “child marriages and the betrothal of young girls before the age of puberty,” though further explanation of what these unions are was omitted from this Convention [31]. A 1965 follow-up to this convention, the Recommendation on Consent to Marriage, Minimum Age for Marriage, and Registration of Marriages, is the first document to explicitly define “full age” as 15 years [32], establishing a legal benchmark for who is eligible to marry among signatories.

More recent international agreements further create legal frameworks for defining marriage. The International Covenant on Economic, Social and Cultural Rights (ICESCR) and the International Covenant on Civil and Political Rights (ICCPR), both drafted in 1966, provide additional guidelines for protecting human rights, particularly for women and children, though these were not enforceable until 1976 [33, 34]. Article 23 of the ICCPR calls for marital union among those who consent and are of “marriageable age.” Other rights protected, such economic and social exploitation of young people (ICESCR, Article 10) and education (ICESCR, Article 13), begin to form a context in which attention must be paid to unions that violate these rights. The 1981 Convention on the Elimination of All Forms of Discrimination Against Women (CEDAW) articulates that women, especially at younger ages, may be vulnerable in marital practices and prohibits discrimination of women; men and women have differential rights and “the marriage of a child” must be eliminated. More specifically, the Convention calls for countries to set a minimum age of marriage and require formal registration [35].

While marriage may seem clearly defined legally, anthropological and demographic literature reveal that marriage is not a straightforward concept. It is a practice symbolizing a union that can have different meanings in different contexts. Bell describes marriage as “a construction in a social space whose dimensions are defined by an articulation of rights and responsibilities” [36]. “In the structurally simplest case,” Bell writes, “marriage involves the entry of a man into a woman’s domestic unit” [36]. One could argue that in contemporary societies, this “entry” might be more figurative, rather than literal, as marital arrangements can now take place with someone half-way across the world. Moreover, marriage is no longer exclusively considered as male-to-female relationships in all contexts. Regardless, marriage is an institution, or an established interpersonal relationship, that offers rights to those in this bond, and in traditional societies, will often involve others beyond the individuals themselves. The extent and strength of those rights, and subsequent responsibilities, may differ in different societies because rights only exist in the context of relationships with others [36].

Marriage, particularly in traditional societies, is rooted in sociocultural and economic contexts involving the joining of two families, rather than just two individuals [37, 38]. This union in many societies may have religious roots. One could point toward several religions (e.g., Christianity, Islam, Judaism) for guidance on religious principles that have informed marital practices. As one example, in the context of Hindu religion in India in the 1890s, Roy describes:

[Marriage] is expressly said to be a divine union. Christ said “What God *hath joined* together, let no man put asunder.” We find Solomon calling the wife a “gift from the Lord,” and in the marriage service appointed by the Church of England some one [sic] is required to stand as the donor of the bride, as is the case in every Hindoo marriage. “Marriage,” says an eminent doctor (Hindoo) of law, “is viewed as a gift of the bride by her father or other guardian to the bridegroom.” The marital union is thus a divine union; it is an act of God and not of man. It is apparent that marriage is not a civil contract, and the consent is not the essence of it. The Roman Catholics regard it as a sacrament; so do the Hindoos [13].

Indeed, Roy’s writings even today serve as a reminder that colonial legacies and an increasing mixture of cultures are redefining how many now conceptualize a marriage. A study in Uganda, for example, illuminates that although concepts like “love” and “faithfulness” are often used synonymously with marriage in discourse, the reality of marriage, in practice, may be expressed in different ways [39]. Marriage is perhaps best viewed as a process – a series of events, decisions, and rites – instead of a dichotomous categorization of a person’s relationship status [37].

Importantly, some relationships considered marriage in local contexts may not result in a legal union [37], creating a challenge for how one measures or documents marital status, particularly in research. Common or civil law provide legislation in support of some marriages, such as based on age and consent of the marrying parties, while customary law and religious teachings may allow divergent unions [15]; age at marriage is one way in which these differences manifest. The lack of agreement, even in a single community, of what constitutes a marriage is moreover complicated by a colonial legacy that implemented laws on marriage that directly clashed with customary law [15]. Demographically, perhaps inspired by these challenges, marriage is today conceptualized to include a union through cohabitation. The Demographic and Health Surveys (DHS) program asks women to self-report on their marital status and counts “married women and women living with a partner” as currently married to generate nationally-representative data on marriage estimates [40]. Similarly, the UNICEF Multiple Indicator Clusters Surveys (MICS), another major international source of nationally-representative household data, asks women: “Have you ever been married or lived together with someone as if married?” and synonymously refers to her spouse as “husband/partner” [41]. This conceptualization of marriage adopts an understanding of customary laws and local norms, providing a more comprehensive picture of how individuals themselves view their relationship status.

Definitions of marriage are constructed in different ways, including legally and socioculturally as briefly touched on above. Legal frameworks are perhaps a top-down approach to constructing definitions of marriage and have resulted in broad international policy agreement on the creation of harmonious regional agreements and national legislation. Subsequently, legal definitions contribute to understanding marriage civilly and across geographic boundaries, which has the advantage of being more tangible to conceptualize, practice, and enforce. Yet in societies as complex and multifaceted as those that exist today, where civil law only serves as one influence on how marriage is constructed and understood, marriage cannot be reduced to its legal definition alone. Although understanding marriage from only a legal perspective loses the customary, religious, and broader sociocultural contexts for which individuals in communities may understand and engage in the practice of marriage, it is among the most common ways to define marriage across contexts.

### Parsing out “Child”

The construct of “girl child marriage” also incorporates the concept of a “child,” another ambiguous and difficult term to define. Here, too, international legal frameworks have played an important role in creating shared global norms defining childhood. As early as 1924, the League of Nations, a precursor to the United Nations (UN) established after World War I to maintain world peace, identified children as an important and special population. A first framework was put forth stating that children “have inalienable rights and are not the property of their father”; Sharma and Gupta [42] further point toward the 1959 UN Declaration on the Rights of the Child, the 1979 declaration as the International Year of the Child, and the 1990 World Summit on Children.

The 1990 Convention on the Rights of Child (CRC), a legally-binding international agreement ratified by all but one country, was a major turning point. Article 1 defines that “a child means every human being below the age of eighteen years unless under the law applicable to the child, majority is attained earlier” [43]. The CRC creates a framework that establishes children, based on years of life, as a special population for whom specific rights should be granted and protected, which proposes something unique about younger human beings. However, while the CRC proposes the age of 18 years as a benchmark of adulthood, the age of a child, or a minor, often legally varies across contexts.

An ethical, or moral, argument supports the establishment of a “child-adult” distinction. As Schapiro [44] argues, certain characteristics are ascribed to someone who is a child, which warrants a different treatment or perspective on their actions until they reach the adult threshold; a child, Schapiro proposes drawing from Immanuel Kant, is “undeveloped” and “dependent.” In other words, there is a level of immaturity children demonstrate, resulting in a lack of agency and requiring additional support until they are able to reason and act independently based on these reasons.

Developmentally, a child is understood to achieve biological, cognitive, psychological, and social milestones over time [45]. At different ages, children are expected to crawl then walk, talk, and express themselves; process increasingly complex forms of information; and develop relationships with others. Children are not expected to behave as maturely as adult counterparts or to clearly assert and describe their own identities, which are shaped overtime as the brain continues to develop and individuals interact with their contexts [46, 47]. Psychology theorists, including Sigmund Freud (founder of psychoanalysis and psychosexual development theory), Erik Erikson (founder of psychosocial development theory), and Jean Piaget (founder of cognitive development theory), have written extensively on child development across different domains [46, 48, 49]. Their different perspectives contribute to the position that childhood is a fluid construct evolving in an individual’s early lifetime.

In recent years, a further distinction of adolescence, referring to older children who are still not yet adults, has emerged. Defined by the World Health Organization (WHO) as individuals aged 10 to 19, adolescents biologically and socioculturally occupy a gray space between childhood and adulthood [50]. Only recently have adolescents been recognized as distinct, more mature than children, yet less developmentally advanced than adults physically, cognitively, and socially [51]. The need to recognize older children as adolescents may be rooted in changing sociocultural contexts, with different expectations and norms for young people. As Bearinger and colleagues explain:

First, acknowledging wide cultural variation, adolescents are increasingly delaying marriage—for some, to pursue education or employment options. Urbanisation has an important role in this societal shift. Second, historically, societies expected childbearing to follow shortly after marriage; now norms are shifting towards delayed childbearing. These key changes, which affect all societies by varying degrees, have expanded the gap between puberty and marriage, and between marriage and childbearing [51].

Moreover, interntional agenda-setting organizations such as the WHO and World Bank have further called out youth (10–24 years) and suggested that for health and human development interventions, there is a need to consider young people through the first 8000 days (through age 21) [52]; such guidance has renewed nominclature on the definition of a child.

Importantly, what perhaps all of this points to, defining a “child” has evolved historically and in different spaces and places. These varying ideas of childhood and adolescence are relational concepts and their definitions at a given moment are influenced by culture, history, local ideology, and different levels of law [53]. Macleod explains that childhood “is not a timeless, transcultural phenomenon”; instead, it should be understood “as the product of a number of cultural processes and modernist ideas, which have come to define a specific life stage as different from others and as in need of special treatment” [53]. Moreover, there may not be a clear or single trajectory from childhood to adolescence to adulthood, and trajectories may vary by context.

The fluidity of the concept of childhood, as detailed above, presents a challenge in the universality of the definition of a child. Different stages of human development are often ascribed to age ranges where these stages are commonly observed and achieved, yet it is not difficult to imagine that transition from childhood to adolescence to adulthood varies depending on local context. For example, where completion of secondary school in some communities signifies a transition, age 18 in one setting may be the normal age of this transition while age 24 may be the age in another setting; completing secondary schooling may be a rare event in other settings, which would make that marker of transition near impossible. In recognition of such differences, normative definitions of a child such as in the CRC acknowledge that countries may have their own guidelines that lower (or raise) the legal age of majority [43]; it is also for this reason that social and cultural constructs of childhood must be acknowledged. The concepts of childhood and adolescence explored have in common the implication that young people have basic universal needs, although how these needs show up and how these needs are met may vary by context [54].

In reflecting on the construct of “girl child marriage,” a child cannot be simply viewed as less than adult: small, weak, young, helpless, little, irresponsible. In fact, such stereotypical ideas of a child are, in many cases and contexts, incorrect. Yet a reference to “girl child marriage” or “child marriage” rather than “adolescent marriage,” “teenage marriage,” or even “early marriage” reminds the reader that a protected population, demonstrated to be less developmentally or socially mature than an adult, is the population being addressed. We have proposed the use of this term to explicitly connect this scientific research with the human rights and advocacy discussions, reminding the reader that the focus is on social experiences young women have experienced preceding their adult life.

A child, as referenced in “girl child marriage,” should be understood to consist of individuals within the first couple of decades of life, afforded varying levels of legal responsibility and accountability, biological and sociocultural maturity, and agency in a cultural context at a given moment in time. Definitions of a child often overlap with an adolescent, pointing toward a unique period earlier in an individual’s life that is distinct across legal, biological, and developmental domains. Like the construct of marriage, international legal frameworks setting a child at age 18 – which draw from theories of child development, ethics, and other areas – have been successful in building agreement around a shared definition of a child that can be operationalized more widely, particularly in the context of consent for marriage.

### The need to focus on girls

We have elected to suggest the need to make a distinction between “child marriage” and “girl child marriage,” a departure from most publications by explicitly engaging with the construct of a “girl.” Child marriage, by definition, impacts both boys and girls [55], yet existing research and advocacy emphasize girls affected due to the prevalence of the practice and the intensity of its consequences for this population [8]. Ages at marriage are lower for females, on average, as compared to male counterparts [56]. Currently, no region is on-track to eliminate child marriage by 2030 to achieve SDG 5 Target 3, and nearly 650 million girls and women living today have been affected [1]. If rates of girl child marriage remain unchanged, 12 million girls under age 18 will continue to marry each year, in contrast to the prevalence of child marriage among boys, estimated to be one-fifth the level of girls [57]. Legal ages of marriage in countries often differ between girls and boys. In fact, a review conducted by the World Bank in 2017 identified 17 countries where the legal age for boys was higher than for girls, including Mali (16 years for girls, 18 years for boys), Iran (13 years for girls, 15 years for boys), and India, where the majority of women married before age 18 reside (18 years for girls, 21 years for boys) [58]. Similarly, social expectations on the ideal age of marriage for males and females often differs. An in-depth qualitative study in Guinea revealed that among the 19 participating women married as children, the majority proposed that the ideal age of marriage for women was younger than that of men [59]. Thus, while recent literature overwhelmingly focuses on the causes and consequences of marriage at younger ages for females, their use of the term “child marriage” masks an implicit gendered lens.

Indeed, while not discounting that boys also experience child marriage, the potential severity of its consequences makes a focus on girls important. Married girls and young women were traditionally considered more protected than unmarried counterparts, due to the perceived economic security and reduced risk of sexually-transmitted infections as a result of a perceived reduction in sexual partners [60]. However, increasing research illuminates that married adolescents may experience limited social support, restricted mobility, and lower levels of education [2]. From the perspective of the health consequences a child spouse may encounter, the hypothesized pathways for girls and boys differ. Child marriage and early childbearing are closely linked, sometimes even discussed interchangeably. According to 2010–2017 data, more than three-fourths of adolescent births (age 15–19) occur in the context of marriage, ranging from 77% in Latin America and the Caribbean to over 90% across Asia and North Africa [61]. Childbearing is not known to directly affect the health of boys through biological pathways yet is of the utmost concern for girls who marry. Additionally, the UN General Assembly recognized explicitly the role that gender inequalities play in the causes and consequences of girl child marriage today [62].

The use of the term “girl child marriage,” moreover, makes explicit what other scholars and practitioners who refer only to child marriage often fail to: that a focus on the causes and consequences of child marriage for only the girl-child has implicitly applied a gendered lens. The use of this term makes explicit the assumption of differences between males and females and identifies the population of focus in much research. Krieger explains that a girl-boy distinction in health and medical research requires thinking beyond a biological division and engaging in the socially-constructed nature of gender, a concept only introduced in the 1970s [63]. We further suggest that beyond the experience of marriage, other variables often measured as related to child marriage including education level and wealth are likely shaped by gender norms, in addition to social norms, and that a girl’s value in her household, community, or society more broadly is influenced by gender norms in the context in which she lives. While further interrogation of this important perspective is beyond the scope of this paper, the articulation of “girl child marriage” signals consideration of these socially-constructed norms.

### Overlaps and distinctions with forced and early marriage

Forced marriage refers to a formal union without the free and full consent of both parties [64]. In contrast to definitions of child, or early, marriage, it is not age-bound. To some, child and early marriage are considered forms of forced marriage because in many contexts, a child, by definition, is unable to provide free and full consent. Such perspectives have not yet been universally adopted. Moreover, there are different terminology practices over the use of child marriage, early marriage, and forced marriage. Certain agencies and initiatives use a combination of all three terms [62, 64, 65]. SDG 5 Target 3 calls for the elimination of “all forms of harmful practices including child, early, and forced marriage and female genital mutilation,” yet its measurement of marriage practices is confined to marriage before age 15 and 18, and not forced marriages that may occur after those age groups [66]. In fact, nationally-representative data on forced marriage remains sparse, and the shared grouping of these concepts masks the differences between these concepts.

While forced marriage may more clearly be distinguished from child marriage, the distinction with early marriage is less clear cut due to different interpretations on what “early” means. To some, “early marriage” serves as a euphemism that hides that children, a protected group, are involved in an act of marriage; moreover, the language of “child brides” romanticizes a problematic practice [67]. To others, “early marriage” better captures that the marital union is premature and encompasses an understanding of different legal and cultural concepts of a child that the term “child marriage” misses [68]. Of note, the legal age of majority is 18 years old in only half of the countries in the world [69]; in a country where the age of majority is reached by 16 years, “early marriage” may more accurately capture the prematurity of a union at age 16 or 17, despite that the individual is not legally a child. There are others who would also argue that an early marriage may even occur after the age of 18 if the spouse is not mature physically and/or emotionally [70].

The term “forced marriage” promotes consideration for the reasons a girl marries early, importantly providing additional context for not only who is affected but why. While acknowledging the variability of legal and social constructs, we propose the language of girl child marriage, or child or early marriage *among girls and women* synonymously, to focus on marriages where the girl-child spouse is below the age of 18. We adopt the convention on the synonymous use of early marriage, defined by UNICEF as “the marriage of children and adolescents below the age of 18” to convey the prematurity of the union [15]. We argue that forced marriage, in its full totality, must be explored separately from girl child marriage and that more research on forced marriage, and its relationship to girl child marriage, is needed.

### Measuring girl child marriage

With such possible variations in an understanding of girl child marriage, it is likely clear that measurement is imperfect. UN estimates use data from the DHS, UNICEF MICS, and other national surveys measuring child marriage as the “percentage of women 20-24 years old who were first married or in union” before 15 years and 18 years [71]. This convention has been set because the indicator for adolescent girl marriage (marriage among girls ages 15–19 years) extends beyond the definition of a child as under age 18 and because measuring marital status among 15- to 17-year-old girls will miss counting women who marry post-survey but still before age 18 [72]. Prevalence of child marriage is also measured through calculating the median age at first marriage among females, using UNICEF MICS and UN Statistics Division data [73]. Importantly, as earlier discussions on social constructs of marriage point toward, measuring girl child marriage includes both formal and informal unions. To this end, UNICEF has importantly and recently articulated that living informally in union, or cohabitation, raises some of the same concerns as marital unions [57].

Girl child marriage relies on self-reported data, which has raised concerns in its validity. A study in India that compared self-reported age at marriage to two calculated indicators – derived by comparing current age to months in marriage as well as current age to months since menarche and marriage – concluded that self-reported age at marriage was an adequate measure in the absence of additional data [74]. Additionally, in many countries, lack of birth registration (confirming the age of the spouse) and lack of marriage registration (confirming a civil marriage and date) complicate a more nuanced perspective of early marital experiences. Consequently, national marriage registries may severely underestimate the number of marriages considered to include girl spouses.

Finally, although we acknowledge the convention of using self-reported data from DHS and UNICEF MICS to measure girl child marriage as a union before age 18, based on available data, we further acknowledge aspirations for future measures. Presumably, a binary cutoff of 18 years omits important information about the consequences of marriage at age 15 or age 12; consequently, several quantitative research studies look at age categories and how they relate to different socioeconomic and health outcomes. Others have recommended more complex indices that adapt from poverty measurement [75].^3^ We encourage, in future efforts, more locally-driven research to better understand and measure girl child marriage from the perspective of those most affected, including through the use of more qualitative methods. We also call for attention toward reaching the existing global benchmarks set in SDG 5 while further creating measures for success beyond martial age alone in order to target progress on gender inequalities that underly why girl child marriage poses human rights, health, and socioeconomic challenges.

## Conclusions

We have presented a theoretical argument defining and deconstructing “girl child marriage.” In doing so, we have articulated consideration for concepts of marriage, children, and gender, which we hope will inform future work particularly impacting the millions of women and girls today who have married before the age of 18. Just as the construct of girl child marriage has evolved over time, we posit that it will continue to evolve.

Additionally, the use of different terms in different contexts may take on a range of meanings at a point in time. To focus only on “early marriage” would prove challenging both quantitatively and qualitatively due to a lack of consistency in how “early” is defined; as we discussed in this paper, “early” referred to age for some while it referred to maturity (independent of age) for others, and it is sensitive to a given context. To focus only on “forced marriage” may result in measuring a different construct and would be challenging due to a dearth of nationally-representative data capturing the extent to which girls’ and women’s marriages are forced, as determined by legal definition and girls and women’s experience; additionally, as we previously called for, more research is needed to better understand forced marriage. To use “adolescent marriage” or “teenage marriage” could result in narrowing the breadth of individuals who marry at younger ages, even in advance of entering adolescence or the teenage years.

Our intent is to encourage more intentional use of language in global public health research, which we argue is lacking to-date. We hope this encourages global public health researchers to engage with the broader social, economic, political, cultural, and historical dimensions of key concepts examined and measured.

## Data Availability

Not applicable.

## Abbreviations

CEDAW: Convention on the Elimination of All Forms of Discrimination Against Women
CRC: Convention on the Rights of Child
DHS: Demographic and Health Surveys
Forum: Forum on Marriage and the Rights of the Children
ICRW: International Center for Research on Women
ICESCR: International Covenant on Economic, Social, and Cultural Rights
ICCPR: International Covenant on Civil and Political Rights
ICDP: International Conference on Population Development
IPPF: International Planned Parenthood Federation
MICS: Multiple Indicator Clusters Surveys
NPR: National Public Radio
SDG: Sustainable Development Goal
UNICEF: United Nations Children’s Fund
UNFPA: United Nations Population Fund
U.S.: United States
WHO: World Health Organization

## Footnotes

1 Similarly, in England, advocacy in 1929 resulted in raising the minimum age of marriage. However, according to the UK Parliament, the minimum age was raised to 16 years for both girls and boys, where it remains, with parental consent, today [16].

2 Personal communication after preparing this manuscript clarified that her intent was to ensure child marriage was recognized as a gendered issue that disproportionately affected girls to capture that her research focused only consequences for females, rather than males, in those analyses.

3 Nguyen and Wodon [75] drew from poverty indices to propose a headcount index of child marriage, equal to the number of girls married below the legal age of marriage divided by the total female population (married and unmarried); a child marriage gap, which accounts for the gap between a girl’s marital age and the legal age of marriage; and a squared child marriage age gap, which would place additional weight on girls who married much earlier than the legal age.

## References

[1] UNICEF. Child Marriage: Latest trends and future prospects [Internet]. 2018 [cited 2020 Jun 5]. Available from: https://data.unicef.org/resources/child-marriage-latest-trends-and-future-prospects/.

[2] UNFPA. State of the world population 2016 [Internet]. New York, New York: UNFPA; 2016. Available from: http://www.unfpa.org/sites/default/files/sowp/downloads/The_State_of_World_Population_2016_-_English.pdf.

[3] UNICEF. A profile of child marriage and early unions in Latin America and the Caribbean [Internet]. 2019 [cited 2020 Jun 5]. Available from: https://www.unicef.org/lac/media/8256/file/Profile%20of%20Child%20Marriage%20in%20LAC.pdf.

[4] Koski A, Heymann J (2018). Child marriage in the United States: how common is the practice, and which children are at greatest risk?. Perspect Sex Reprod Health.

[5] De Groot R, Kuunyem MY, Palermo T (2018). Child marriage and associated outcomes in northern Ghana: a cross-sectional study. BMC Public Health.

[6] Raj A, Boehmer U. Girl child marriage and its association with national rates of HIV, maternal health, and infant mortality across 97 countries. Violence Against Women [Internet]. 2013;19(4):536–51 Available from: http://journals.sagepub.com/doi/10.1177/1077801213487747. 10.1177/1077801213487747 23698937

[7] John NA, Edmeades J, Murithi L (2019). Child marriage and psychological well-being in Niger and Ethiopia. BMC Public Health.

[8] UNICEF. Ending child marriage: progress and prospects [Internet]. 2014 [cited 2020 Jun 5]. Available from: https://data.unicef.org/resources/ending-child-marriage-progress-and-prospects/.

[9] Belanger A. A new study on child marriage is changing the conversation [Internet]: Teen Vogue; 2018. [cited 2020 June 5]. Available from: https://www.teenvogue.com/story/new-study-child-marriage-changing-conversation.

[10] Belanger A. Child marriage in the United States, explained [Internet]: Teen Vogue; 2017. [cited 2020 Jun 5]. Available from: https://www.teenvogue.com/story/child-marriage-in-the-united-states-explained.

[11] Reiss F. Despite progress, child marriage is still legal in all 50 states [Internet]. The New York Times - The Opinion Pages. 2017 [cited 2020 Jun 5]. Available from: https://kristof.blogs.nytimes.com/2017/07/26/despite-progress-child-marriage-is-still-legal-in-all-50-states/.

[12] Adair K, Sinclair S. A photographer gives cameras to child brides. Their images are amazing. [Internet]. NPR - Goats and Soda. 2016 [cited 2020 Jun 5]. Available from: https://www.npr.org/sections/goatsandsoda/2016/10/08/491797689/a-photographer-gives-cameras-to-child-brides-their-images-are-amazing.

[13] Roy RC. Child marriage in India. Source: The North American Review [Internet]. 1888;147(383):415–23 Available from: https://www.jstor.org/stable/25101631.

[14] Bhandarkar RG. History of child-marriage [Internet]. Vol. 47, Zeitschrift der Deutschen Morgenländischen Gesellschaft. Harrassowitz Verlag; 1893 [cited 2020 Jun 11]. p. 143–56. Available from: https://www.jstor.org/stable/43362297.

[15] Black M, Haeri V, Moodie N. Early marriage: child spouses (Vol. 7) [Internet]. Florence, Italy; 2001. Available from: www.unicef-icdc.org.

[16] The law of marriage [Internet]. UK Parliament. [cited 2020 Jun 26]. Available from: https://www.parliament.uk/about/living-heritage/transformingsociety/private-lives/relationships/overview/lawofmarriage-/.

[17] Smyth G. Child marriage in England. Med World [Internet]. 1957;87(4):349–50. Available from: http://www.ncbi.nlm.nih.gov/pubmed/13477067 [cited 2020 Jun 28]. 13477067

[18] Gruenberg E. [Child marriage in Israel]. Harefuah [Internet]. 1955;48(2):33–34. Available from: http://www.ncbi.nlm.nih.gov/pubmed/14353380 [cited 2020 Jun 28]. 14353380

[19] Mati JK, Mbugua S, Ndavi M (1984). Control of cancer of the cervix: feasibility of screening for premalignant lesions in an African environment. IARC Sci Publ.

[20] United Nations. Report of the International Conference on Population and Development [Internet]. 1995 [cited 2020 Jun 27]. Available from: https://www.un.org/en/development/desa/population/events/pdf/expert/27/SupportingDocuments/A_CONF.171_13_Rev.1.pdf.

[21] Burns JF. Though illegal, child marriage is popular in part of India. The New York Times [Internet]. 1998 [cited 2020 Jun 27]; Available from: https://www.nytimes.com/1998/05/11/world/though-illegal-child-marriage-is-popular-in-part-of-india.html.

[22] Mathur S, Greene M, Malhotra A. Too young to wed: the lives, rights, and health of young married girls [Internet]. Washington, D.C.; 2003 [cited 2020 Jun 11]. Available from: https://www.icrw.org/wp-content/uploads/2016/10/Too-Young-to-Wed-the-Lives-Rights-and-Health-of-Young-Married-Girls.pdf.

[23] Somerset C. Early marriage: whose right to choose? [Internet]. 2000 [cited 2020 Jun 11]. Available from: www.khubmarriage18.org/sites/default/files/228.pdf.

[24] Jensen R, Thornton R. Early female marriage in the developing world. Gender Dev [Internet]. 2003;11(2):9–19 Available from: http://www.jstor.org/stable/4030636 [cited 2020 June 27].

[25] UNICEF. Early marriage: a harmful traditional practice [Internet]. New York; 2005 [cited 2020 June 27]. Available from: https://www.unicef.org/publications/files/Early_Marriage_12.lo.pdf.

[26] International Planned Parenthood Federation and the Forum on Marriage and the Rights of Women and Girls. Ending child marriage: a guide for global policy action. [Internet]. London, England; 2006. Available from: https://www.unfpa.org/sites/default/files/pub-pdf/endchildmarriage.pdf.

[27] Raj A (2010). When the mother is a child: the impact of child marriage on the health and human rights of girls. Arch Dis Childhood [Internet].

[28] Schwelb E (1963). Marriage and human rights. Am J Comp Law [Internet].

[29] Sarich J, Olivier M, Bales K. Forced marriage, slavery, and plural legal systems: an African example. Hum Rights Q [Internet]. 2016;38(2):450–76 Available from: https://muse.jhu.edu/article/617748 [cited 2020 June 26].

[30] UN General Assembly. Universal Declaration of Human Rights [Internet]. 1948. Available from: https://www.un.org/en/universal-declaration-human-rights/.

[31] UN General Assembly (1962). Convention on Consent to Marriage, Minimum Age for Marriage [Internet].

[32] UN General Assembly (1965). Recommendation on Consent to Marriage, Minimum Age for Marriage [Internet].

[33] UN General Assembly (1966). International Covenant on Civil and Political Rights [Internet].

[34] UN General Assembly (1966). International Covenant on Economic, Social and Cultural Rights [Internet].

[35] UN General Assembly (1979). Convention on the Elmination of All Forms of Discrimination Against Women [Internet].

[36] Bell D (1997). Defining marriage and legitimacy. Curr Anthropol [Internet].

[37] Haberland N, Chong E, Bracken H (2003). Married adolescents: an overview. WHO/UNFPA/Population Council Technical Consultation on Married Adolescents [Internet].

[38] Chinwuba NN. Interaction of customs and colonial heritage. Their impact on marriage and children in Nigeria. Anthropos. 2016;111(1):49–68.

[39] Agol D, Bukenya D, Seeley J, Kabunga E, Katahoire A. Marriage, intimacy and risk of HIV infection in south west Uganda. Afr J Reprod Health. 2014;18(4):86–94. 25993749

[40] Measure DHS/ICF International. Description of the Demographic and Health Surveys individual recode data file [Internet]. Washington, DC; 2013. [cited 2020 Jun 26]. Available from: https://www.dhsprogram.com/pubs/pdf/DHSG4/Recode6_DHS_22March2013_DHSG4.pdf.

[41] Multiple Indicator Clusters Survey Program. Instructions for reviewers [Internet]. New York; 2019 [cited 2020 Jun 26]. Available from: https://www.google.com/url?sa=t&rct=j&q=&esrc=s&source=web&cd=&cad=rja&uact=8&ved=2ahUKEwicwMfzsaLqAhU5JzQIHXDYD6UQFjABegQIARAB&url=http%3A%2F%2Fmics.unicef.org%2Ffiles%3Fjob%3DW1siZiIsIjIwMTkvMDEvMDcvMTUvMTUvMDMvODMzL01JQ1M2X0luc3RydWN0aW9uc19mb3JfSW50ZXJ2aWV3ZXJzXzIwMTkwMTAyLmRvY3giXV0%26sha%3De3d2c06935199e4c&usg=AOvVaw2i7bb4caAJPZfCWHY2AkbP.

[42] Sharma A, Gupta S. Child’s right to special care. ICCW New Bull [Internet]. 1991;39(3–4):12–5 Available from: https://pubmed.ncbi.nlm.nih.gov/12317284/ [cited 2020 Jun 26]. 12317284

[43] UN General Assembly (1990). Convention on the Rights of the Child [Internet].

[44] Schapiro T. What is a child? Ethics. 1999;109(4):715–38.

[45] Efevbera Y, McCoy DC, Wuermli AJ, Betancourt TS. Integrating early child development and violence prevention programs: a systematic review. New Dir Child Adolesc Dev. 2018;8:27–54. 10.1002/cad.20230 29537183

[46] Erikson E (1950). Childhood and society.

[47] Bronfenbrenner U (1977). Toward an experimental ecology of human development. Am Psychologist [Internet].

[48] Freud S. Three Essays on the Theory of Sexuality (1905). The standard edition of the complete psychological works of Sigmund Freud, volume VII (1901–1905): A case of hysteria, three essays on sexuality and other works; 1953. 123–246.

[49] Piaget J. The origins of intelligence in children. M. Cook, Trans. New York: International Universities Press, Inc.; 1952.

[50] World Health Organization. Adolescent health [Internet]. [cited 2020 Jun 26]. Available from: https://www.who.int/health-topics/adolescent-health/#tab=tab_1.

[51] Bearinger L, Sieving R, Ferguson J, Sharma V (2007). Global perspectives on the sexual and reproductive health of adolescents: patterns, prevention, and potential. Lancet [Internet].

[52] Bundy D, de Silva N, Horton S, Jamison D, Patton G. Disease control priorities, third edition, volume 8: child and adolescent health and development [Internet], Washington, DC: The World Bank; 2017. Available from: http://elibrary.worldbank.org/doi/book/10.1596/978-1-4648-0423-6.

[53] Macleod C (2003). Teenage pregnancy and the construction of adolescence in South Africa. Childhood.

[54] Actions for the Rights of the Children. ARC resource pack: a capacity-building tool for child protection in and after emergercies [Internet]. 2015 Mar [cited 2020 Jun 26]. Available from: https://resourcecentre.savethechildren.net/library/arc-resource-pack-actions-rights-children.

[55] Strochlic N. The sad hidden plight of child grooms. Daily Beast [Internet]. 2014; [cited 2020 Jun 26]; Available from: https://www.thedailybeast.com/the-sad-hidden-plight-of-child-grooms.

[56] United Nations Department of Economic and Social Affairs - Population Division. World marriage data 2017 [Internet]. [cited 2020 Jun 26]. Available from: https://www.un.org/en/development/desa/population/theme/marriage-unions/WMD2017.asp.

[57] UNICEF. Child marriage - UNICEF DATA [Internet]. [cited 2020 Jun 5]. Available from: https://data.unicef.org/topic/child-protection/child-marriage/.

[58] Khohkhar T. The age of marriage & legal gender differences - 3 charts for International Women’s Day [Internet]. Data Blog. 2017; [cited 2020 Jun 26]. Available from: https://blogs.worldbank.org/opendata/age-marriage-legal-gender-differences-3-charts-international-women-s-day.

[59] Efevbera Y. Why do young girls marry? A qualitative study on drivers of girl child marriage in Conakry, Guinea. Vancouver: Women Deliver; 2019.

[60] Bruce J, Clark S. Including married adolescents in adolescent reproductive health and HIV/AIDS policy. Geneva: WHO/UNFPA/Population Council Technical Consultation on Married Adolescents. Geneva: The World Bank Group; 2003.

[61] United Nations Department of Economic and Social Affairs - Population Division. DHS and MICS microdata sets on adolescent births. 2020.

[62] UN General Assembly 71st session. 71/175. Child, early, and forced marriage [Internet]. Geneva, Switzerland; 2017 [cited 2020 Jun 26]. Available from: https://www.un.org/en/ga/search/view_doc.asp?symbol=A/RES/71/175&referer=http://www.un.org/en/ga/71/resolutions.shtml&Lang=E.

[63] Krieger N (2003). Genders, sexes, and health: what are the connections—and why does it matter?. Int J Epidemiol [Internet].

[64] Glinski AM, Sexton M, Meyers L. Child, Early, and forced marriage resource guide task order [Internet]. Washington, DC; 2015. Available from: https://www.usaid.gov/documents/1865/child-early-and-forced-marriage-resource-guide.

[65] UN General Assembly Human Rights Council 24th session. 24/...Strengthening efforts to prevent and eliminate child, early and forced marriage : challenges, achievements, best practices and implementation gaps [Internet]. 2013 [cited 2020 Jun 26]. Available from: https://www.refworld.org/docid/53bd11df4.html.

[66] UN General Assembly 70th session. 70/1. Transforming our world: the 2030 agenda for sustainable development [Internet]. Geneva, Switzerland; 2015 [cited 2020 Jun 26]. Report No.: A/Res/70/1. Available from: https://www.un.org/ga/search/view_doc.asp?symbol=A/RES/70/1&Lang=E.

[67] Nour N (2009). Child marriage: a silent health and human rights issue. Rev Obstet Gynecol [Internet].

[68] Myers J. Untying the knot. Exploring early marriage in fragile states. World Vision. 2013;.

[69] Youth Policy Labs. Age Matters! Age-related barriers to service acces and the realisation of rights for children, adolescents and youth [Internet]. 2016 Dec [cited 2020 Jun 26]. Available from: https://agemattersnow.org/downloads/YPL_Age_Matters_Final_Report_Oct2016.pdf.

[70] UNFPA. Child marriage - frequently asked questions [Internet]. 2017 [cited 2020 Jun 26]. Available from: https://www.unfpa.org/child-marriage-frequently-asked-questions#what%20is%20the%20difference%20between%20child%20marriage,%20early%20marriage%20and%20forced%20marriage?.

[71] UNICEF. The state of the world’s children 2017: children in a digital world [Internet]. New York; 2017. Dec [cited 2020 Jun 26]. Available from: https://www.unicef.org/publications/index_101992.html.

[72] Loaiza Sr. E, Wong S. Marrying too young [Internet]. New York; 2012 [cited 2020 Jun 26]. Available from: https://www.unfpa.org/sites/default/files/pub-pdf/MarryingTooYoung.pdf.

[73] Aspen Planning and Evaluation Program, Girls Not Brides. Measuring progress: recommended indicators for Girls Not Brides members working to address child marriage [Internet]. London; 2015 [cited 2020 Jun 26]. Available from: https://www.girlsnotbrides.org/wp-content/uploads/2014/12/GNB_Full-List-of-Indicators_August-2015_Final1.pdf.

[74] Raj A (2017). Validity of self-reported age at marriage in rural India. Int J Gynecol Obstet [Internet].

[75] Nguyen M, Wodon Q. Measuring child marriage. Econ Bull. 2012;32(1):398–411.

